# Disclosing the functional changes of two genetic alterations in a patient with Chronic Progressive External Ophthalmoplegia: Report of the novel mtDNA m.7486G>A variant

**DOI:** 10.1016/j.nmd.2017.11.006

**Published:** 2018-04

**Authors:** Mafalda Bacalhau, Marta Simões, Mariana C. Rocha, Steven A. Hardy, Amy E. Vincent, João Durães, Maria C. Macário, Maria João Santos, Olinda Rebelo, Carla Lopes, João Pratas, Cândida Mendes, Mónica Zuzarte, A. Cristina Rego, Henrique Girão, Lee-Jun C. Wong, Robert W. Taylor, Manuela Grazina

**Affiliations:** a FMUC – Faculty of Medicine, University of Coimbra, Coimbra, Portugal; b CNC – Center for Neuroscience and Cell Biology, Laboratory of Biochemical Genetics, University of Coimbra, Coimbra, Portugal; c Wellcome Centre for Mitochondrial Research, Institute of Neuroscience, The Medical School, Newcastle University, Newcastle Upon Tyne, UK; d CHUC – Neurology Department of Coimbra University Hospitals, Coimbra, Portugal; e CNC – Center for Neuroscience and Cell Biology, University of Coimbra, Coimbra, Portugal; f IBILI – Institute for Biomedical Imaging and Life Sciences, University of Coimbra, Coimbra, Portugal; g Mitochondrial Diagnostic Laboratory, Baylor College of Medicine, Houston, USA

**Keywords:** CPEO, mtDNA common deletion, mt-tRNA variant (m.7486G>A), Bioenergetic dysfunction, Translation defect

## Abstract

Chronic Progressive External Ophthalmoplegia (CPEO) is characterized by ptosis and ophthalmoplegia and is usually caused by mitochondrial DNA (mtDNA) deletions or mt-tRNA mutations. The aim of the present work was to clarify the genetic defect in a patient presenting with CPEO and elucidate the underlying pathogenic mechanism. This 62-year-old female first developed ptosis of the right eye at the age of 12 and subsequently the left eye at 45 years, and was found to have external ophthalmoplegia at the age of 55 years. Histopathological abnormalities were detected in the patient's muscle, including ragged-red fibres, a mosaic pattern of COX-deficient muscle fibres and combined deficiency of respiratory chain complexes I and IV. Genetic investigation revealed the “common deletion” in the patient's muscle and fibroblasts. Moreover, a novel, heteroplasmic mt-tRNA^Ser(UCN)^ variant (m.7486G>A) in the anticodon loop was detected in muscle homogenate (50%), fibroblasts (11%) and blood (4%). Single-fibre analysis showed segregation with COX-deficient fibres for both genetic alterations. Assembly defects of mtDNA-encoded complexes were demonstrated in fibroblasts. Functional analyses showed significant bioenergetic dysfunction, reduction in respiration rate and ATP production and mitochondrial depolarization. Multilamellar bodies were detected by electron microscopy, suggesting disturbance in autophagy. In conclusion, we report a CPEO patient with two possible genetic origins, both segregating with biochemical and histochemical defect. The “common mtDNA deletion” is the most likely cause, yet the potential pathogenic effect of a novel mt-tRNA^Ser(UCN)^ variant cannot be fully excluded.

## Highlights

• Novel mt-tRNA^Ser(UCN)^ (m.7486G>A) variant found in CPEO patient with 4,977 bp deletion.
• The variant located in the anticodon loop meets the pathogenicity criteria.
• Both genetic defects segregate with the biochemical phenotype in muscle.
• Assembly impairment of MRC complexes was detected.
• Mitochondrial translation defect and bioenergetic dysfunction were revealed.

## 1 Introduction

One of the most common presentations of mitochondrial DNA (mtDNA)-associated disease in adulthood is Chronic Progressive External Ophthalmoplegia (CPEO), which is characterized by progressive paralysis of the extraocular muscles (EOMs) leading to ptosis and impaired eye movement (ophthalmoplegia) [1].

CPEO is commonly caused by either primary mitochondrial genetic defects such as single, large-scale mtDNA deletions [1], [2], [3], [4], [5], [6] and mt-tRNA point mutations [7], [8], [9], [10], [11], [12], [13], [14], [15], [16], [17], [18], [19], or multiple mtDNA deletions, which are secondary to primary mutations in nuclear genes responsible for the maintenance of mitochondrial genome integrity [20], [21]. The single 4,977 bp deletion, known as the “common deletion”, is the most frequent genetic defect found in patients with CPEO [22], [23].

Muscle biopsies of CPEO patients typically comprise subsarcolemmal accumulation of abnormal mitochondria known as ragged-red fibres (RRF) and a mosaic pattern of COX-deficient fibres showing abnormal COX activity. Mitochondrial dysfunction occurs in a tissue where the number of mutated mtDNA accumulates and exceeds a biochemical threshold (threshold effect), which has been shown to vary for different types of mutation, namely 50–80% for single, large-scale mtDNA deletions and 70–95% for tRNA point mutations [24], [25]. Currently, more than half of disease-related mtDNA point mutations have been reported within mt-tRNA genes that cause defective translation and, consequently, combined respiratory chain deficiency. Any deletion that eliminates an mt-tRNA gene causes the same impairment of overall mitochondrial-encoded proteins.

Studies reporting single, large-scale mtDNA deletions have rarely provided functional evidences of the genetic defect [3], [5], [26], [27], [28], [29], [30], [31]; therefore, deeper investigation regarding the affected cellular mechanisms is still needed.

The aim of the present work was to: (i) clarify the molecular genetic defect and (ii) elucidate which cellular mechanisms are affected in a patient presenting with CPEO that harbours a novel mt-tRNA^Ser(UCN)^ variant (m.7486G>A, ClinVar accession number: SCV000492500) in addition to the “common deletion”.

## 2 Patient and methods

### 2.1 Case report

The patient is a 62-year-old Portuguese Caucasian female followed at the Neurology Department of the Coimbra Hospital University Centre. The first clinical signs started at the age of 12 years old with slowly progressive ptosis of the right eyelid and later involvement of the other eyelid at the age of 45 years old. At this point, she was observed in the context of a corneal ulcer and complaints that she had “difficulties keeping her eyes open”. The disease maintained a slow progression and she was diagnosed with CPEO at the age of 55 years old, with the neurological examination revealing severe bilateral eyelid ptosis, ophthalmoplegia and dysphonia. There was no reported family history of CPEO or any other mitochondrial disorder.

Biological samples (peripheral blood, skin and muscle biopsies) were collected from the patient investigated in the present study during diagnostic investigation. There were no samples available from maternal relatives.

The DNA samples of 200 adult healthy subjects of the same ethnic background were used as controls.

Informed consent was obtained from the participants, as recommended by the local Ethics Committee (CE-032/2014), following the Tenets of the Helsinki Declaration.

### 2.2 Histology, histochemistry and quadruple immunofluorescence in muscle

Routine histological (Haematoxylin & Eosin – H&E, modified Gomori trichrome staining) and histochemical (cytochrome *c* oxidase [COX], succinate dehydrogenase [SDH], and sequential COX/SDH) analysis of the patient's skeletal muscle was performed, by following standard methods [32].

Quadruple immunofluorescence was performed on cryosectioned patient skeletal muscle (n = 1022) using antibodies detecting subunits of OXPHOS complexes: anti-NDUFB8 for Complex I (CI) and anti-COXI for Complex IV (CIV), as described previously [33]. Mitochondrial mass was quantified using an antibody to Porin and the myofibre boundaries were labelled with the antibody to Laminin.

### 2.3 Skin derived cultured fibroblasts

Fibroblasts from skin biopsies of the patient and three healthy Portuguese individuals (control group), without clinical evidence of mitochondrial disease, were grown in complete medium supplemented with 20% FBS (Gibco, Life Technologies) and antibiotics.

### 2.4 Genetic investigation in different tissues

Total DNA was extracted from several tissues including blood, dermal fibroblasts and muscle homogenate according to standard protocols [34], [35].

Individual COX-positive and COX-deficient muscle fibres were isolated by laser microcapture, using a PALM Laser Capture Microdissection system, and lysed to obtain total cellular DNA, as previously described [36]. The stained sections were then used for single-cell molecular analyses.

#### 2.4.1 Whole mitochondrial genome sequencing

The presence of sequence variants and rearrangements were detected by subjecting the patient's mitochondrial genome to next generation sequencing (NGS) in all available tissues, enriched by a single amplicon long-range PCR followed by massively parallel sequencing [37], using the HiSeq2000 platform (Illumina technology).

Haplogroup of patient was determined using the Haplogrep^®^ tool [38].

#### 2.4.2 *In silico* analysis

The *in silico* analysis included the evolutionary conservation of the *MT-TS1* gene (mt-tRNA^Ser(UCN)^) from mtDNA of different species, according to the proposed consensus panel [39] using ClustalOmega|EBI^®^ [40]. The location of the m.7486G>A sequence variation in the cloverleaf structure of mt-tRNA^Ser(UCN)^ was verified from Mamit-tRNA^®^ database [41].

#### 2.4.3 Screening for mtDNA rearrangements

The presence of rearrangements in mtDNA was confirmed in DNA derived from muscle homogenate, using an established triplicate long range PCR approach for large-scale multiple and single mtDNA deletions, amplifying approximately 10, 13 and 16 kb of the mtDNA in three separate reactions. For ~10 kb amplification PCR forward-F (m.6122–6139) and reverse-R (m.16133–16153) primers were used. For ~13 kb amplification PCR F (m.13965–13984) and R (m.129-110) primers were used and for ~16 kb amplification PCR F (m.1157–1167) and R (m.19-1) were employed. Cycling conditions were: 94 °C, 2 min; 35 cycles of 94 °C, 30 sec and 65 °C, 16 min; 72 °C 16 min. Amplifying PCR products were separated in 0.7% agarose gels.

#### 2.4.4 Single fibre studies

The m.7486G>A mt-tRNA mutation load was assessed by pyrosequencing technology, in all available tissues and in individual COX-positive and COX-deficient muscle fibres. The PyroMark Assay Design Software v.2.0. (Qiagen) was used to design locus-specific PCR and sequencing primers for the m.7486G>A variant (biotinylated forward primer: m.7466–7485; reverse primer: m.7583–7600; sequence primer: m.7488–7502) and pyrosequencing was performed on the Pyromark Q24 platform, according to the manufacturer's protocol. Pyromark Q24 software was used to quantify the m.7486G>A heteroplasmy levels by directly comparing the peak heights of both wild-type and mutant nucleotides at this position [42].

The multiplex *MTND1/MTND4* real-time PCR assay was performed using DNA from individual COX-deficient and COX-positive isolated muscle fibres and muscle homogenate and the mtDNA deletion level was calculated from the proportion of wild-type (*MTND4*) to total (*MTND1*) copy number by the established ΔΔC_t_ method [43]. PCR amplification was completed in a 25 µl reaction in triplicate for each sample, with each plate containing a serial dilution of p7D1 plasmid for standard curve generation, as reported previously [44].

### 2.5 Mitochondrial respiratory chain (MRC) enzymatic activity and respiratory rate evaluation

Spectrophotometric determination of the catalytic activity of the MRC complexes and segments was performed as previously described [45].

Oxygen consumption rate (OCR) was measured in adherent fibroblasts with a XF24 Extracellular Flux Analyser (Seahorse Bioscience, Billerica, MA, USA), essentially as described by Zhang and co-workers [46].

Each of the three controls' and patient's fibroblasts were seeded in XF24 cell culture microplates (Seahorse Bioscience). OCR was measured under basal conditions, and after sequentially adding oligomycin, FCCP and rotenone plus antimycin A were also added. Results were expressed as pmol of O_2_ per minute per mg of protein, and allowed the evaluation of the following bioenergetics parameters: basal respiration (BR), maximum respiration (MR), spare respiratory capacity (SRC), ATP production capacity (APC) and proton leak (PL). All determinations were performed in 9–12 replicates for each sample.

### 2.6 Analysis of mitochondrial membrane potential

Alterations in mitochondrial membrane potential **(ΔΨ_m_)** were determined using 1.5 µM of the cationic fluorescent probe rhodamine 123 (Molecular probes, Invitrogen) in Krebs medium, for 1 h, at 37 °C. Basal fluorescence (λ = 540 nm for excitation and λ = 590 nm for emission) was measured using a Microplate Spectrofluorometer Gemini EM (Molecular Devices, USA) at 37 °C, for 5 min, followed by the addition of 2 µg/ml oligomycin and 2 µM FCCP. Results were expressed as the difference between the basal fluorescence values and the increase in rhodamine 123 fluorescence levels following addition of oligomycin plus FCCP.

### 2.7 Relative quantification of MCR complexes

Samples were processed according to the protocol described elsewhere [47]. Briefly, following supplementation of samples with BN-sample buffer, the molecular weight marker (NativeMARK Unstained Protein Standard, Life Technologies) and 30 µg of samples were loaded into polyacrylamide gels and run at 80V at 4 °C. The gel containing the proteins of interest was eletrotransferred to a PVDF membrane (Hybond P 0.5 µm, Amersham) for 2 h at 0.2A, at 4 °C. Afterwards, membranes were incubated with monoclonal primary antibodies [anti-NDUFA9 for CI; anti-SDHA for Complex II (CII); anti-UQCRC2 for Complex III (CIII); anti-COX IV for CIV; and anti-ATP5A for Complex V (CV)]. Membranes were incubated with the anti-mouse HRP-conjugated secondary antibody solution. Subsequently, detection was carried out using a chemiluminescence substrate (Clarity Western ECL Substrate, Bio-Rad), through the ChemiDocTM XRS+ System (Bio-Rad). Protein band intensities were calculated by Quantity One® 1-D software (Bio-Rad) from at least 3 independent experiments. Relative semi-quantitation of each complex assembled was performed in comparison to CII levels.

### 2.8 Transmission electron microscopy (TEM)

Muscle fibres from a fresh biopsy were fixed with 4% glutaraldehyde in 0.2M sodium cacodylate buffer (pH 7.2) for 4 h. After rinsing twice in the same buffer, fibres were post-fixed in 1% osmium tetroxide for 2 h. Following rinsing in buffer, samples were then dehydrated in a graded ethanol series (75–100%) before being embedded in Epoxy resin (Fluka Analytical). Semi-thin sections (2 µm) were obtained and stained with toluidine blue for light microscopy in order to identify the area of interest.

Fibroblasts were collected and centrifuged at 775 x*g* for 5 min. The supernatant was discarded and pellet cells were fixed with 2.5% glutaraldehyde in 0.1M sodium cacodylate buffer (pH 7.2) supplemented with 1 mM calcium chloride for 2 h. Following rinsing in the same buffer, post-fixation was performed using 1% osmium tetroxide for 1 h. After rinsing twice in buffer, and distilled water and, 1% aqueous uranyl acetate was added to the cells, for contrast enhancement during 1 h in the dark. After rinsing in distilled water, samples were dehydrated in a graded acetone series (30–100%), impregnated and embedded in Epoxy resin (Fluka Analytical).

Finally, for both preparations, ultrathin sections (70 nm) were mounted on copper grids (300 mesh) and stained with lead citrate 0.2%, for 7 min. Observations were carried out on a FEI-Tecnai G2 Spirit Bio Twin at 100 kV and images were acquired using the software AnalySIS 3.2.

### 2.9 Statistical analysis

Data were analysed using GraphPad Prism version 5.00 software for Windows, San Diego, California, USA. Normality tests were applied in order to assure the Gaussian distribution of the results. Statistical analysis of the patient's vs. controls' results was assessed by a Student's *t*-test (or nonparametric Mann-Whitney test).

Statistical significance is represented as * for 0.050 ≥ *p* > 0.010, ** for 0.010 ≥ *p* > 0.001 and *** for *p* ≤ 0.001.

## 3 Results

### 3.1 Histochemistry and quadruple immunofluorescence presented evidences for mitochondrial dysfunction in muscle

**Fig. 1:**
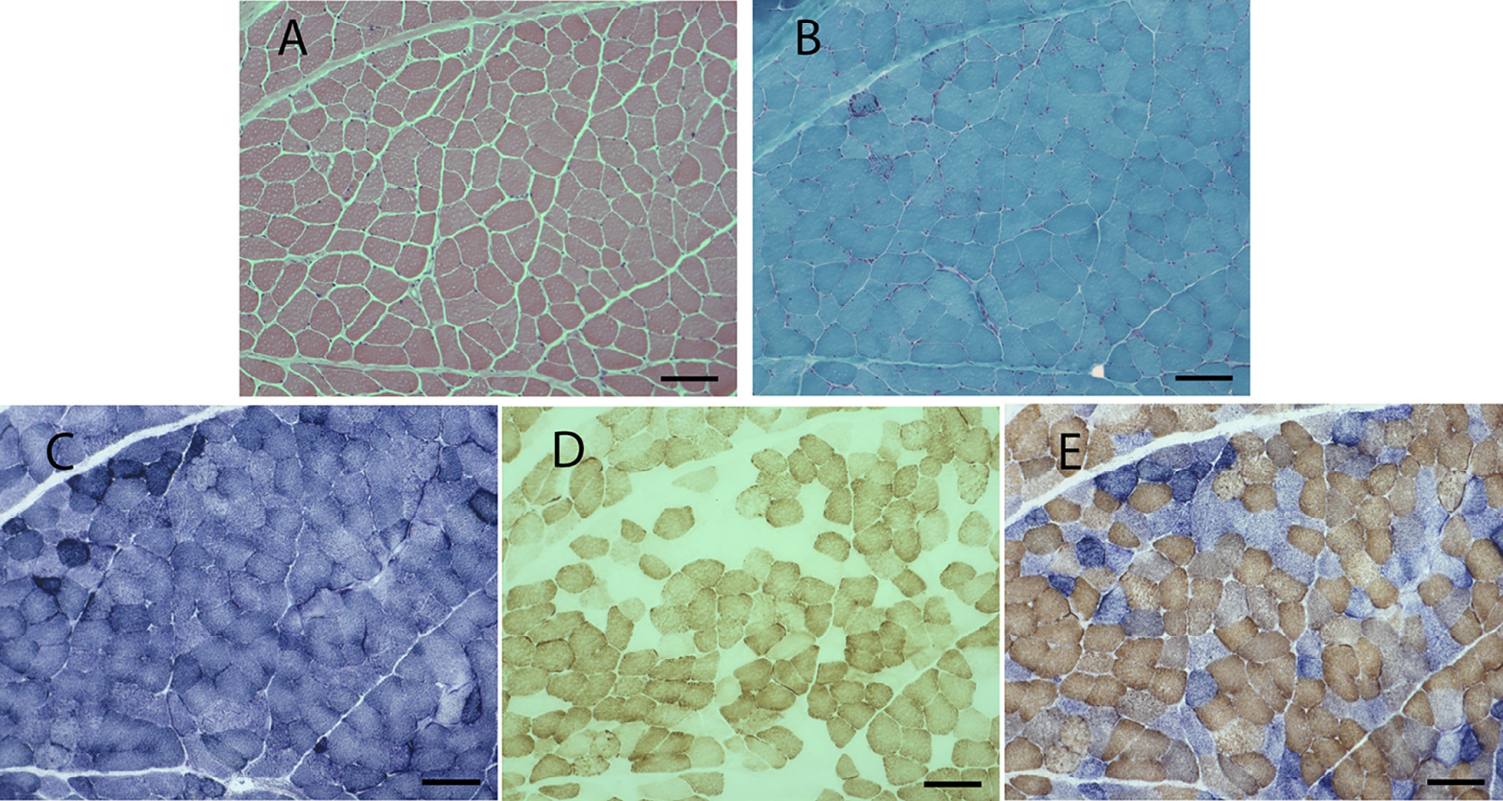
Histopathological features associated with mtDNA disease in patient's skeletal muscle. (A) H&E staining showing general muscle morphology; (B) modified Gomori trichrome staining highlighting muscle RRF; (C) SDH staining, which reveals subsarcolemmal accumulation of mitochondrial activity; (D) COX-deficient fibres and COX-positive fibres; (E) sequential COX/SDH histochemistry emphasising individual COX-deficient fibres which retain SDH activity. Scale bar: 100 µm.

**Fig. 2:**
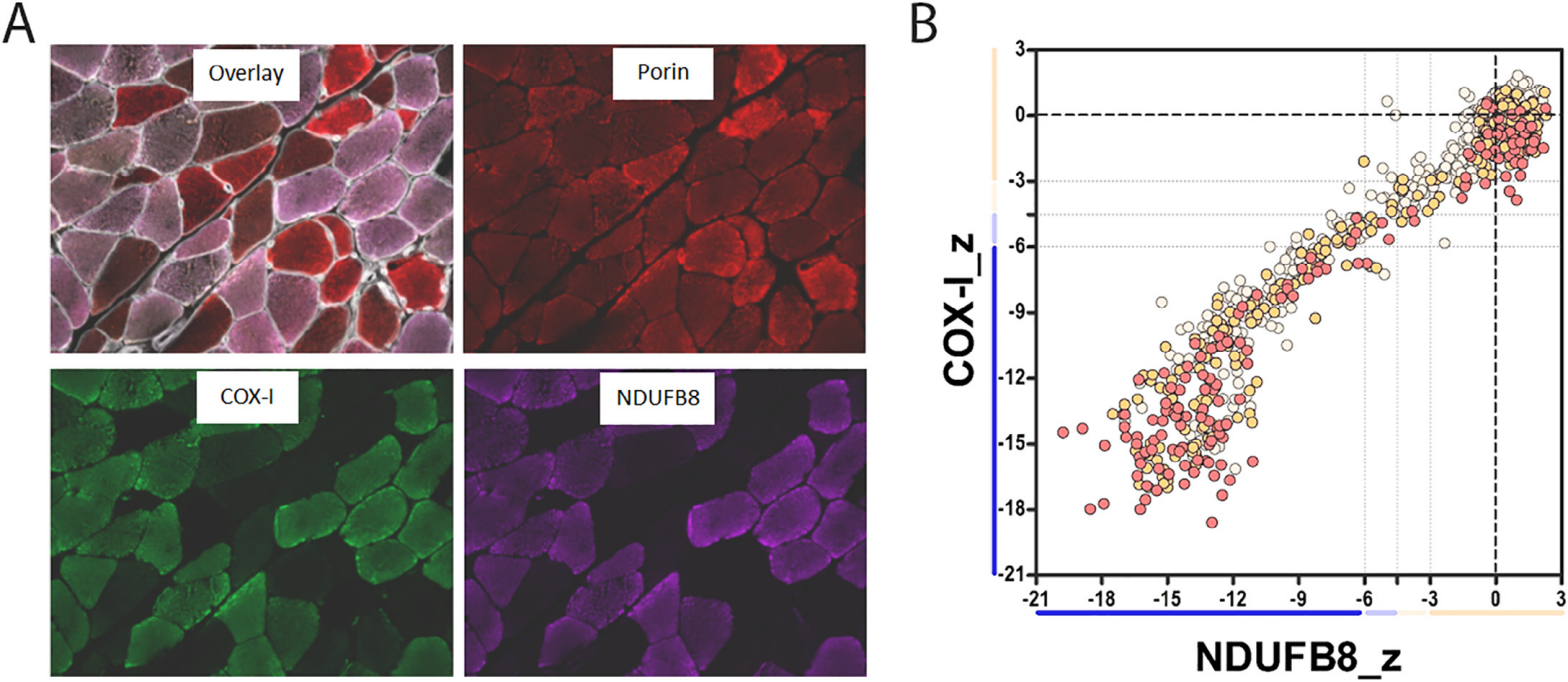
Mitochondrial respiratory chain profile in patient's biopsy. (A) Representative image of quadruple immunofluorescence (laminin – white (405 nm), NDUFB8 – purple (647 nm), COX-I – green (488 nm) and porin – red (546 nm)) performed in patient's muscle section. (B) Mitochondrial respiratory chain profile showing complex I, complex IV and porin levels in patient (n = 1022 fibres). Each dot represents an individual muscle fibre colour coded according to its mitochondrial mass (very low: blue, low: light blue, normal: light orange, high: orange and very high: red). Thin black dashed lines indicate the SD limits for the classification of fibres, lines next to x and y axis indicate the levels of NDUFB8 and COX-I respectively (beige: normal, light beige: intermediate(+), light blue: intermediate(−) and blue: negative), bold dashed lines indicate the mean expression level of normal fibres.

Histological and histochemical examination of cryosectioned patient skeletal muscle revealed the presence of approximately 10% RRF as a result of mitochondria subsarcolemmal accumulation (Fig. 1A,B) and a significant proportion (40%–50%) of COX-deficient fibres (Fig. 1C–E). Assessment by quadruple immunofluorescence (Fig. 2A) showed an equal down regulation (43%) of both CI and CIV levels in individual fibres (Fig. 2B); the MRC profile (Fig. 2B) was similar to the profiles previously reported for single, large-scale deletion [33].

### 3.2 Genetic investigation revealed the presence of the “common deletion” and a novel mt-tRNA^Ser(UCN)^ variant at high levels in muscle

The entire mitochondrial genome analysis by NGS revealed previously reported polymorphisms and a novel heteroplasmic variant in the mt-tRNA^Ser(UCN)^ (m. 7486G>A, ClinVar accession number: SCV000492500) (Supplementary material – Table S1) in the patient's muscle homogenate, fibroblasts and lymphocytes. Besides, a heteroplasmic single, large-scale deletion was detected in muscle homogenate and fibroblasts of patient, but not in blood, with the sequence breakpoints determined as 8482–13447, corresponding to the “common deletion”.

The previous analysis determined that the patient belongs to the haplogroup H+16311.

Heteroplasmy quantification of the mt-tRNA^Ser(UCN)^ variant (m.7486G>A) was obtained by pyrosequencing in fibroblasts (~10%) and muscle homogenate (~50%).

**Fig. 3:**
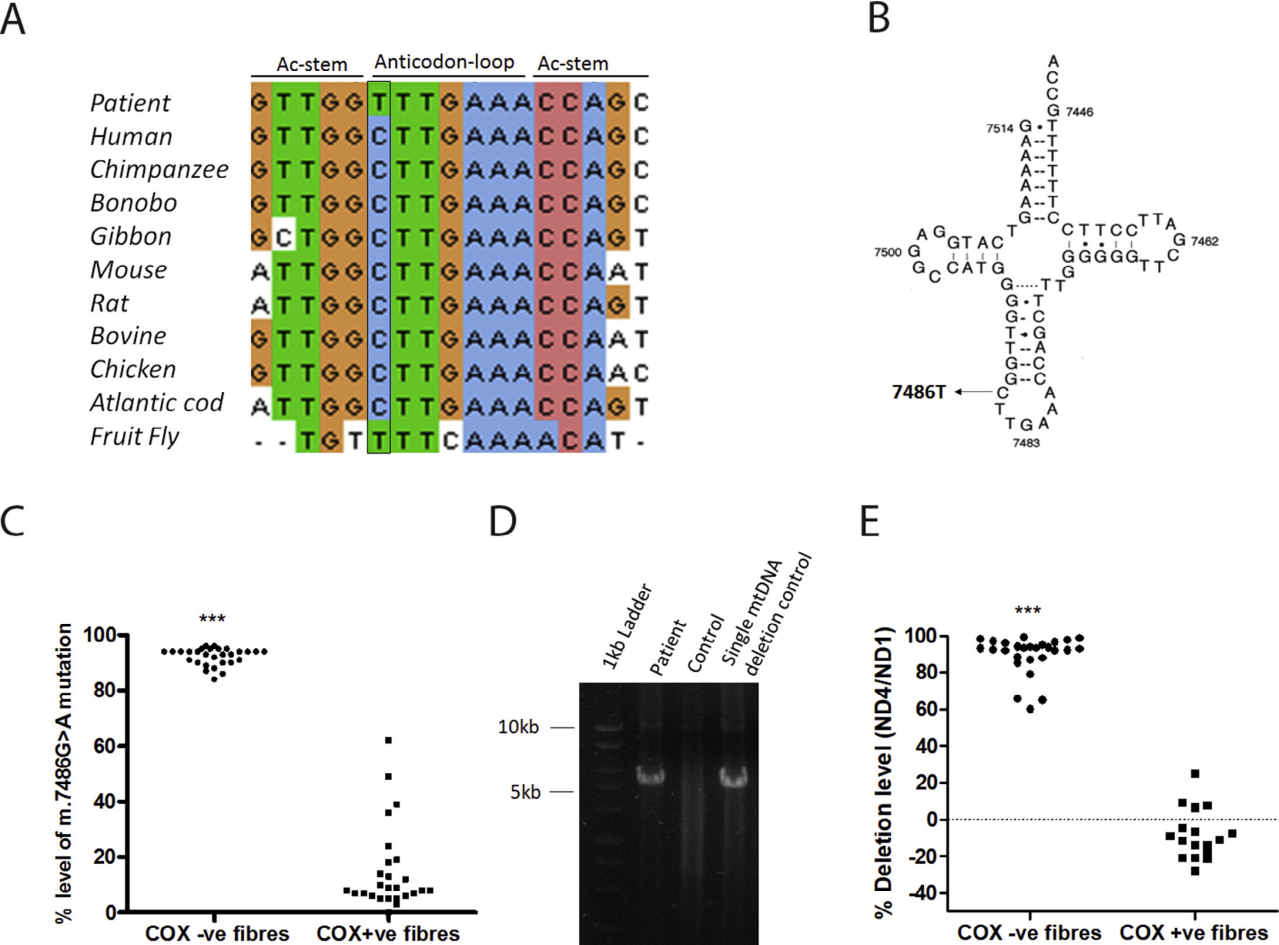
Genetic investigation of the “common deletion” and the novel mt-tRNA^S^*^er(UCN)^* variant. (A) Evolutionary conservation of m.7486G>A change in mt-tRNA*^Ser(UCN)^* in different species. (B) Proposed secondary structure of mt-tRNA*^Ser(UCN)^*. The gene is encoded in the light strand and thus the base change at position 7486 is shown as C to T. (C) Single-fibre analysis by pyrosequencing of the m.7486G>A mutation in individual COX-deficient and COX-positive muscle fibres, showing a clear segregation of high mutation load with the biochemical defect. Mann-Whitney test, ***p <* *0.0001. (D) Long-range PCR for detection of the “common deletion”, amplifying approximately 10 kb of the mtDNA, producing a product size of approximately 5 kb. (E) Single fibre study for the presence of the “common deletion” in individual COX-deficient and COX-positive muscle fibres after Real-time PCR analysis, showing a clear segregation of high mutant load with the biochemical defect. Mann-Whitney test, ***p <* *0.0001.

The sequence variation was present at residue 32 in the anticodon loop of mt-tRNA^Ser(UCN)^ affecting a 3-methylcytidine post-transcriptional position (Fig. 3A). The evolutionary conservation showed that cytosine is conserved throughout evolution (Fig. 3B). Moreover, the same alteration was not detected in the DNA samples of 200 controls screened by Sanger sequencing (data not shown).

Long-range PCR for ~10 kb amplification presented a product size band with ~10 kb (*wild type*) and also ~5 kb, confirming the presence of the single, large-scale deletion (Fig. 3D), which is in agreement with the deletion size detected by NGS.

Single muscle fibre analysis revealed significantly higher levels of the heteroplasmic variant m.7486G>A (***p <* *0.0001) in COX-deficient fibres (92.24 ± 0.58%, n = 29), compared to COX-positive fibres (14.96 ± 2.98%, n = 26) (Fig. 3C). Also, the common deletion load showed significantly higher levels (***p <* *0.0001) in COX-deficient fibres (89.58 ± 2, n = 27) and the absence of deletion in most of COX-positive muscle fibres (n = 17), (Fig. 3E). Accordingly, muscle homogenate presented 57% to 61% of deletion in the three independent experiments performed.

### 3.3 Assembly of MRC complexes was impaired

**Fig. 4:**
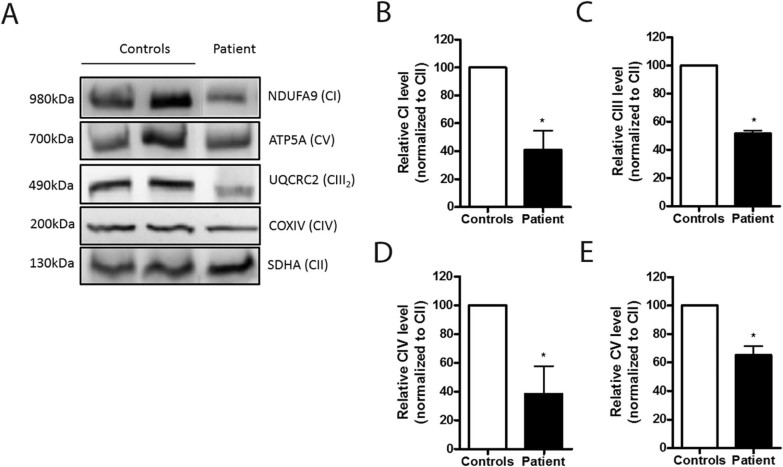
(A) Blue-native PAGE followed by immunoblot with antibodies directed against specific individual subunits, in order to quantify the fully assembled OXPHOS complexes. Complex II was used as loading control. (B) Relative fully assembled complex I level, *p = 0.0162; (C) Relative fully assembled complex III level, *p = 0.0127; (D) Relative fully assembled complex IV level, *p = 0.0357; (E) Relative fully assembled complex V level, *p = 0.0226. The analysis was performed using Mann-Whitney test. Results are representative of the mean ± SEM based on at least three independent experiments in duplicate. C – Complex.

A statistically significant reduction in the amount of fully-assembled MRC complexes containing mtDNA-encoded proteins was observed in the patient's fibroblasts compared to controls (Fig. 4A), suggesting a mitochondrial translation defect. The results are similar for the four complexes with mitochondrial subunits: CI (Fig. 4B, *p = 0.0162), CIII (Fig. 4C, *p = 0.0127), CIV (Fig. 4D, *p = 0.0357) and CV (Fig. 4E, *p = 0.0216).

### 3.4 Mitochondrial dysfunction detected in patient's cells: bioenergetics impairment, membrane depolarization and morphological changes

Assessment of MRC enzymatic activity (Supplementary material – Table S4) in muscle homogenate revealed a reduction in CIV activity (44.8% of controls). Also, CV activity was diminished in muscle and skin fibroblasts (69% and 66% of the controls' mean, respectively).

**Fig. 5:**
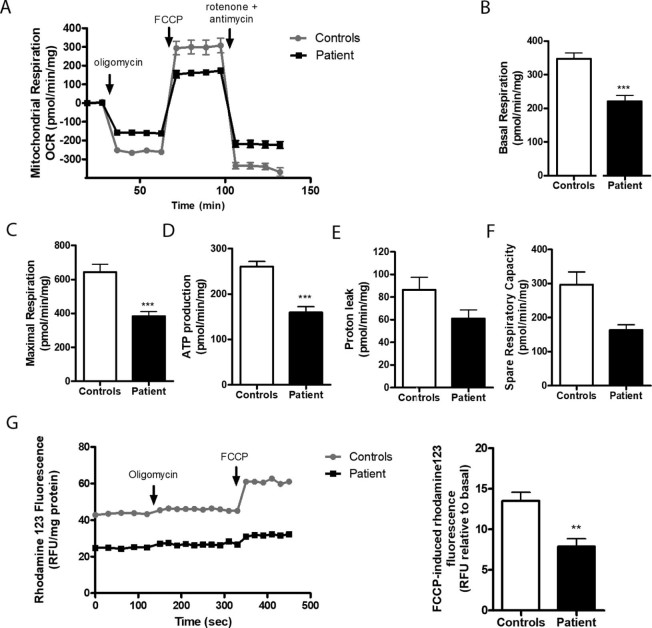
Determination of parameters for mitochondrial function. (A) Mitochondrial Respiration Profile; (B) Basal respiration, Unpaired t-test, ***p = 0.0008; (C) Maximal respiration, Mann Whitney test, ***p = 0.0008; (D) ATP production, Unpaired t-test, ***p <* *0.0001; (E) Proton leak; (F) Spare respiratory Capacity; (G) Mitochondrial membrane potential measurement in patient's skin fibroblasts and control group, after maximal depolarization with oligomycin plus FCCP (Unpaired Student's t-test: **p = 0.0084). Results are the mean ± SEM of three independent measurements run in triplicates.

The OCR results showed a significant reduction in mitochondrial respiration of the patient's cells compared to controls (Fig. 5A–F); there were evident differences in basal respiration (***p = 0.0008), maximal respiration (***p = 0.0008) and ATP production (***p <* *0.0001).

Moreover, the addition of the uncoupler FCCP in cells pre-exposed to oligomycin (to inhibit CV) caused a rise in rhodamine 123 fluorescence that was significantly lower in the patient's cells than in controls' cells (**p = 0.0084) (Fig. 5G), suggesting mitochondrial membrane depolarization in patient's fibroblasts.

**Fig. 6:**
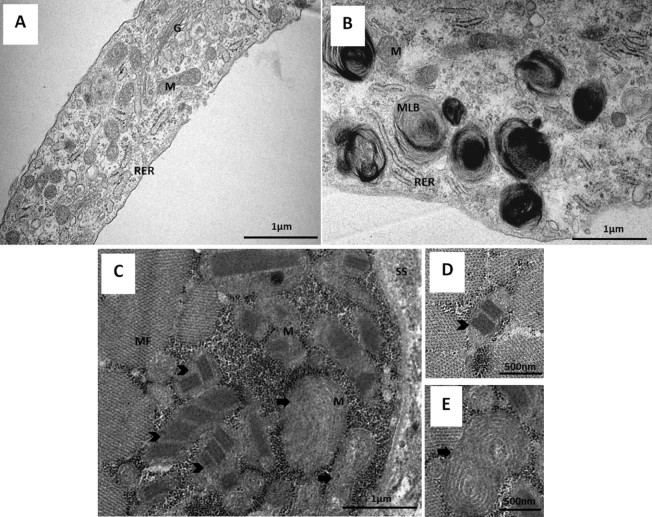
Ultrastructural study of primary fibroblasts and skeletal muscle by transmission electron microscopy. (A) Control's fibroblasts, (B) Patient's fibroblasts showing multilamellar bodies (MLB), (C-E) Myofibres of patient's skeletal muscle in the transverse plane, presenting abnormal mitochondria with paracrystalline inclusions (arrowhead) and concentric cristae (arrows). G – Golgi, M – Mitochondrion, RER – Rough Endoplasmic Reticulum, MF – myofibrils, SS – Subsarcolemmal space.

Remarkable morphological and ultrastructural differences were observed between control (Fig. 6A) and patient (Fig. 6B) skin fibroblasts, namely the presence of large multilamellar bodies (MLB) (Fig. 6B). Mitochondrial hyperproliferation, enlarged mitochondria and mitochondria presenting structural alterations of cristae such as paracrystalline inclusions and concentric “onion-shaped” cristae, were observed in patient's muscle (Fig. 6C–E).

## 4 Discussion

In the present report, we describe a patient with histological and histochemical changes in muscle that were indicative of OXPHOS dysfunction and mitochondrial disease. Both immunofluorescence and MRC evaluation revealed decreased levels and activity of multiple complexes in muscle sections and homogenate, respectively, likely due to defective mitochondrial translation. Deep genetic investigation allowed the identification of the “common deletion” in muscle and fibroblasts, and a novel mt-tRNA^Ser(UCN)^ variation (m.7486G>A) in muscle, fibroblasts and blood. For single-fibre studies, we have used the same muscle fibres to quantify the levels of the mtDNA deletion and the tRNA variant. Both presented high levels within the isolated COX-deficient fibres and low levels within COX-positive fibres. Consequently, it was not possible to clarify the segregation of the tRNA variant alone, within the COX-deficient fibres, because the biochemical segregation was similar for both alterations. The percentage of deleted molecules is probably enough to cause a clinical phenotype, while the presence of a novel heteroplasmic variant in the anticodon loop of the mt-tRNA^Ser(UCN)^ was also relevant.

There are numerous examples of disease-causing mutations in mt-tRNA genes and more than 20 variants, in 13 different mt-tRNA genes, have been reported in association with CPEO and/or myopathy [48]. Three of these have been reported in the *MT-TS1* gene (encoding mt-tRNA^Ser(UCN)^): m.7506G>A [49], m.7451A>T [50] and m.7458G>A [51] have all been associated with PEO. Nevertheless, the interpretation of pathogenicity for mtDNA variants is complex and challenging. The pathogenic variants in mt-tRNA genes may impair transcription termination and tRNA maturation, reduce the aminoacylation, abolish post-transcriptional modification of tRNA, decrease the binding to translation factors or the mitoribosome, alter the structure, perturb the stability, and disturb codon reading, ultimately leading to loss of function [52], [53]. The novel variation m.7486G>A affects a highly conserved nucleotide and alters the first base of the anticodon loop adjacent to the anticodon stem (position 32 of mt-tRNA^Ser(UCN)^), disturbing a 3-methylcytidine (m^3^C) post-transcriptional modification position [53], [54], [55], [56], [57]. Little is known about the function of this type of modification, although a role in accurate codon recognition and translation efficiency has been suggested [58], [59]. Thus, the m.7486G>A variation could affect the conformation of the anticodon loop by creating a U32 pair with A38, that may disturb the interaction with mitoribosome, thus reducing the contact time for codon recognition. Similar defects in the conformation of the anticodon loop were suggested for the m.7480T>C variation, reported as pathogenic and causing mitochondrial myopathy, located in the anticodon loop of mt-tRNA^Ser(UCN)^ at position 38 (opposite to 32) [60]. The novel variation herein reported was identified (about 90%) in COX-deficient fibres of the affected tissue (muscle), segregating with the biochemical phenotype but maintaining the mt-tRNA^Ser(UCN)^ steady-state levels (Supplementary material – Figure S1). Accordingly, there is a possibility that the novel sequence variant might compromise mt-tRNA^Ser(UCN)^ anticodon function by disrupting the correct binding of mitoribosome and/or the recognition of the amino acid sequence, leading to consequences in mitochondrial translational accuracy, without any effect on mt-tRNA^Ser(UCN)^ stability.

According to the scoring criteria [61] for characterisation of the pathogenicity for the novel m.7486G>A variant, the score totals 11 points, out of 20, as follows: (1) the variant was heteroplasmic in different tissues (2 points); (2) the base is conserved throughout evolution (2 points); (3) there was a strong histochemical evidence of mitochondrial disease (2 points); (4) biochemical defects were detected in complexes I, III or IV (2 points); and (5) the variant segregated with the biochemical phenotype (3 points). Some of these criteria were certainly influenced by the presence of the “common deletion”. However, the specific criterion for the segregation with the biochemical defect suggested that the sequence variation is “probably pathogenic”.

The defective mitochondrial translation is predicted to be the primary consequence of mutations affecting mt-tRNA genes, leading to OXPHOS deficiency. The “common deletion” would be expected to have a similar detrimental effect on translation of all subunits encoded by mitochondrial genome. Therefore, the most likely genetic cause of CPEO in this patient is the “common deletion”, even though the m.7486G>A sequence variation holds a huge pathogenic potential associated to the disease.

Since the first report [22], the consequences of the “common deletion” have been characterized [3], [5], [26], [27], [28], [29], [30], namely the decrease in MRC complexes activity [3], [5], [27], [29], mitochondrial protein synthesis [28], mitochondrial membrane potential and ATP synthesis [30]. In fact, functional evidences were gathered, demonstrating the inability to produce mtDNA-encoded proteins, leading to incomplete fully assembled OXPHOS complexes and resulting in electron transport deficiency, inefficient respiration and depolarization of mitochondrial membrane, confirming previous studies.

Concerning ultrastructural alterations, the most relevant finding in fibroblasts was the presence of MLB, which could be interpreted as an expression of cellular damage, suggesting alterations of autophagy, similarly to the hypothesis proposed by Signorini and colleagues in skin fibroblasts of patients affected with Rett Syndrome [31] and Thomas and co-workers in fibroblasts of patients presenting Spinocerebellar Ataxia, autosomal recessive 20 [62]. The accumulation of abnormal mitochondria in the patient's skeletal muscle, presenting paracrystalline inclusions and concentric cristae, reinforces the mitochondrial dysfunction observed in this tissue, as previously described for myopathy caused by single, large-scale mtDNA deletion or mt-tRNA mutations [63]. These results strongly suggest that defects in the proteolytic systems, namely mitophagy, responsible for the elimination of damaged or dysfunctional mitochondria, can be implicated in the disease.

In conclusion, we describe a patient presenting with CPEO who harbours two genetic alterations contributing to the clinical phenotype: a single, large-scale mtDNA deletion and a novel mt-tRNA sequence variant. Both alterations were detected by NGS. However, due to technical limitations, more specific analyses were performed in order to confirm and quantify the level of heteroplasmy of the mtDNA deletion and the tRNA variant. Since both alterations show high levels in muscle, appear to segregate with the biochemical defect and may promote inefficient mitochondrial translation, it is difficult to assign the isolated impact of the novel mt-tRNA sequence variation, thus demonstrating the inherent difficulties in proving the pathogenicity of novel or rare mtDNA variants***.*** The “common deletion” is the most likely genetic cause. However, the potential pathogenic effect of the novel mt-tRNA^Ser(UCN)^ sequence variation cannot be ignored, and it may be valuable in evaluating further cases. Similar patients (i.e. presenting with two mtDNA genetic defects) are reported in the literature; one patient presented with myopathy and autoimmune polyendocrinopathy due to a single mtDNA deletion co-existing with the pathogenic m.3243A>G variant [64]; another patient presented with limb-girdle myopathy associated with two, heteroplasmic mtDNA sequence variants [65]; together with the case we report here, these reinforce the idea that multiple mtDNA variants may act synergistically to influence clinical phenotype.

## Appendix Supplementary material

Fig. S1 Representation of mt-tRNA*^Ser(UCN)^* steady-state levels by high-resolution northern blot in skeletal muscle, without significant differences between patient and controls. Fig. S1

Fig. S2 Glycolytic function measured for patient and controls' cells. Data are representative of the mean ± SEM. (A) Acidification profile; (B) Glycolysis; (C) Glycolytic Capacity; (D) Glycolytic Reserve. Fig. S2

Table S1 Mitochondrial sequence variations detected by whole mitochondrial genome sequencing in the patient presenting CPEO. Table S1

Table S2 List of the 513 nuclear genes related to mitochondrial function and structure sequenced by Next Generation Sequencing (Mitome500 panel, Unpublished, Lee-Jun C. Wong). Table S2

Table S3 List of the sequence variations detected in nuclear genes in patient's samples, after filtering for variant calling. Table S3

Table S4 Assessment of enzymatic activity of the mitochondrial respiratory chain complexes. Results are expressed as nmol of substrate per min per mg of protein normalized to Citrate Synthase. Patient values are presented as the mean of replicates. Control values are shown as mean ± SD. Table S4

Appendix S1 Methods and results description. Appendix S1
