# Supplementary figures and images for: Disclosing the functional changes of two genetic alterations in a patient with Chronic Progressive External Ophthalmoplegia: Report of the novel mtDNA m.7486G>A variant
Source: Neuromuscul Disord. 2018 Apr;28(4):350–60. doi: 10.1016/j.nmd.2017.11.006 (PMC5952895; doi:10.1016/j.nmd.2017.11.006)

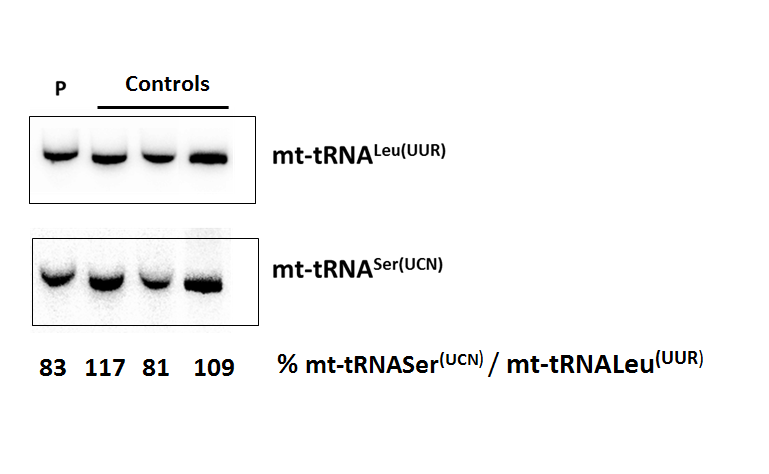

Supplement: Fig. S1 — Representation of mt-tRNASer(UCN) steady-state levels by high-resolution northern blot in skeletal muscle, without significant differences between patient and controls. [file mmc1.zip › mmc1.tif]

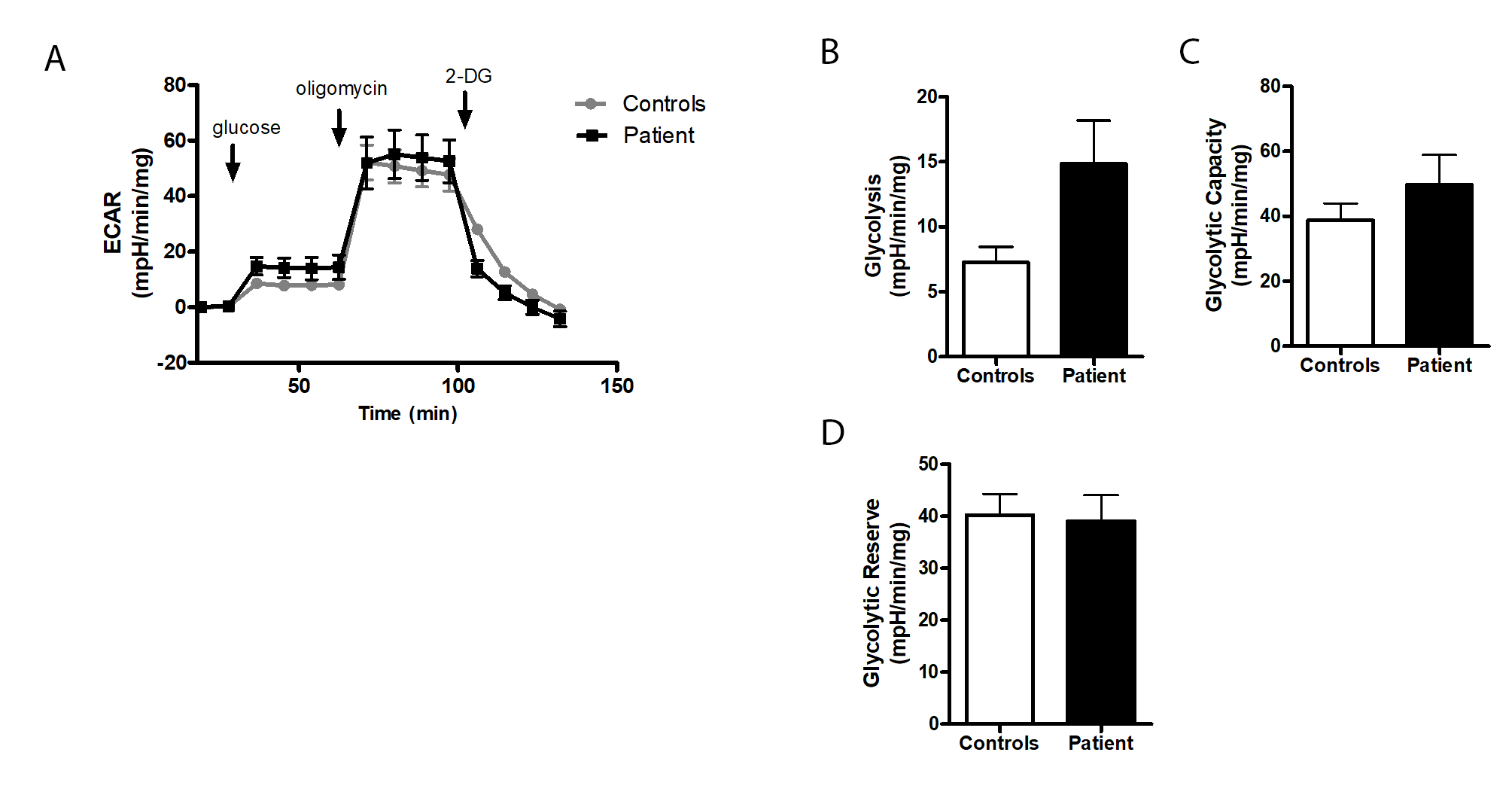

Supplement: Fig. S2 — Glycolytic function measured for patient and controls' cells. Data are representative of the mean ± SEM. (A) Acidification profile; (B) Glycolysis; (C) Glycolytic Capacity; (D) Glycolytic Reserve. [file mmc2.zip › mmc2.tif]
