# Supplementary material for: Disclosing the functional changes of two genetic alterations in a patient with Chronic Progressive External Ophthalmoplegia: Report of the novel mtDNA m.7486G>A variant
Source: Neuromuscul Disord. 2018 Apr;28(4):350–60. doi: 10.1016/j.nmd.2017.11.006 (PMC5952895; doi:10.1016/j.nmd.2017.11.006)
Supplement: Appendix S1 — Methods and results description. [file mmc7.docx]

**Disclosing the functional changes of two genetic alterations in a patient with Chronic Progressive External Ophthalmoplegia: report of the novel mtDNA m.7486G>A variant**

Mafalda Bacalhau^a,b^, Marta Simões^b^, Mariana C. Rocha^c^, Steven A. Hardy^c^, Amy E. Vincent^c^, João Durães^d^, Maria C. Macário^d^, Maria João Santos^a,b^, Olinda Rebelo^d^, Carla Lopes^e^, João Pratas^b^, Cândida Mendes^b^, Mónica Zuzarte^f^, A. Cristina Rego^a,e^, Henrique Girão^a,f^, Lee-Jun C. Wong^g^, Robert W. Taylor^c^, Manuela Grazina^a,b,1^

*^a^FMUC – Faculty of Medicine, University of Coimbra, Coimbra, Portugal*

*^b^CNC – Center for Neuroscience and Cell Biology, Laboratory of Biochemical Genetics, University of Coimbra, Coimbra, Portugal*

*^c^Wellcome Centre for Mitochondrial Research, Institute for Neuroscience, Medical School, Newcastle University, Newcastle Upon Tyne, UK*

*^d^CHUC – Neurology Department of Coimbra University Hospitals, Coimbra, Portugal*

*^e^CNC – Center for Neuroscience and Cell Biology, University of Coimbra, Coimbra, Portugal*

*^f^IBILI – Institute for Biomedical Imaging and Life Sciences, University of Coimbra, Coimbra, Portugal*

*^g^Mitochondrial Diagnostic Laboratory, Baylor College of Medicine, Houston, USA*

^1^Corresponding author: mgrazina.fmuc@gmail.com

Patient and Methods

**1. Confirmation of the mt-tRNA novel sequence variation**

Automated Sanger sequencing analysis was performed, according to the manufacturer's instructions (3130 ABI Prism sequencing system), using BigDye^®^ Terminator Ready Reaction Mix v3.1 (Applied Biosystems), for investigating the region 7241-7644, in the available tissues of the patient. The genetic screening of the 200 controls’ samples was carried out by the same method to check for the presence of the novel mtDNA sequence variation (m.7486G>A) detected in the patient.

**2. Quantification of mt-tRNA*^Ser(UCN)^*** **steady-state level**

The steady-state levels of mt-tRNA*^Ser(UCN)^* were determined using the RNA extracted from muscle specimens of the patient and unaffected controls by high-resolution Northern blot analysis, as described(45), using probes for mt-tRNA*^Ser(UCN)^* and mt-tRNA*^Leu(UUR)^*. Following PhosphorImager analysis, the radioactive signal for the mt-tRNA*^Ser(UCN)^* probe was normalized to the mt-tRNA*^Leu(UUR)^* probe.

**3. Nuclear Panel investigation**

To analyse potential nuclear gene involvement, coding exons of 513 (Supplementary material – Table 2) candidate genes related to mitochondrial structure and function were hybridized with customized oligonucleotide probe library, captured and then massively sequenced using the HiSeq2000 platform (Illumina technology^®^).

**4. Glycolytic evaluation by Seahorse Bioscience technology**

By directly measuring the ECAR, the XF Glycolysis Stress Test provides the parameters of glycolytic flux: Glycolysis, Glycolytic Capacity, and Glycolytic Reserve. After ECAR baseline measurements, a saturating concentration of glucose allows to calculate glycolysis under basal conditions. Afterwards, oligomycin was injected, revealing the cellular maximum glycolytic capacity. The difference between Glycolytic Capacity and Glycolysis rate defines Glycolytic Reserve. Data units are mpH (index of the acidification of the medium surrounding the cells as protons are produced and extruded) *per* minute *per* mg of protein.

Results

**1. Quantification of mt-tRNA steady-state level by high-resolution Northern blot**

Concerning the investigation of the processing of mt-tRNA*^Ser(UCN)^*, the steady-state level of this tRNA was determined in the patient’s muscle by Northern blot hybridization. Levels of the mature mt-tRNA*^Ser(UCN)^* transcript were almost unchanged when compared to controls (Supplementary material – Figure 1).

**2. Nuclear genes investigation**

The Mitome500 panel revealed some polymorphisms and unknown variants in heterozygosity (Supplementary material - Table 3). A number of candidate genes involved in mtDNA replication and maintenance, including *POLG*, *TWNK* (formerly *PEO1*), *MPV17*, *TK2*, *SLC25A4* (*ANT1*), *OPA1*, *TYMP*, *DGUOK*, *SURF1* and *RRM2B,* were investigated, but the variants found could not explain the mtDNA rearrangement. Also some genes involved in mitophagy, namely *PINK1* and *PARK2*, did not present variants likely to be deleterious.

**3. Glycolytic evaluation by Seahorse Bioscience Technology**

In order to compensate the reduced mitochondrial respiration rate, glycolysis and glycolytic capacity are enhanced, although not significantly (Supplementary material – Figure 2).
